# CD73 promotes proliferation and migration of human cervical cancer cells independent of its enzyme activity

**DOI:** 10.1186/s12885-017-3128-5

**Published:** 2017-02-15

**Authors:** Zhao-wei Gao, Hui-ping Wang, Fang Lin, Xi Wang, Min Long, Hui-zhong Zhang, Ke Dong

**Affiliations:** Department of Clinical Diagnosis, Tangdu Hospital, Fourth Military Medical University, Xinsi, Road, Xi’an, Shanxi 710038 China

**Keywords:** CD73, Cervical cancer, Proliferation, Migration, Enzyme activity

## Abstract

**Background:**

CD73 has both enzymatic and non-enzymatic functions in cells. As a nucleotidase, CD73 plays its enzymatic function by catalyzing the hydrolysis of AMP into adenosine and phosphate. In addition to this, accumulating data have shown that CD73 is a key regulatory molecule involved in cancer growth and metastasis, but this non-enzymatic function of CD73 in cervical cancer cells has not been well studied.

**Methods:**

CD73 was overexpressed by pcDNA-NT5E expression vector transfection in Hela and SiHa cells. Cell’s proliferation and migration were evaluated by MTT and scratch healing assay. The CD73 specific antagonist -APCP was used to inhibit CD73 enzymatic activity. And the effect of APCP on CD73 activity was determined by high performance liquid chromatography (HPLC). Expression level was assessed by qRT-PCR and western blotting.

**Results:**

In the present study, we used Hela and SiHa cell lines to evaluate the effects of CD73 on cervical cancer cells proliferation and migration, and further explore the potential regulating mechanisms. Our data showed that CD73 overexpression significantly promoted cervical cancer cells proliferation and migration, and this promotive effect was not reverted by blocking CD73 enzymatic activity, both in Hela and SiHa cells. On the other hand, our data also showed that high concentration of adenosine inhibited Hela and SiHa cells proliferation and migration. These results demonstrated that the promotive effect of CD73 on cervical cancer cells proliferation and migration in vitro was independent from its enzymatic activity (i.e. production of adenosine). Furthermore, the expressions of EGFR, VEGF and Akt were significantly increased in CD73 overexpression Hela and SiHa cells.

**Conclusions:**

Our data suggested that CD73 might promote proliferation and migration via potentiating EGFR/Akt and VEGF/Akt pathway, which was independent of CD73 enzyme activity. These data provide a novel insight into the regulating function of CD73 in cancer cells and suggest that CD73 may be promising therapeutic target in cervical cancer.

**Electronic supplementary material:**

The online version of this article (doi:10.1186/s12885-017-3128-5) contains supplementary material, which is available to authorized users.

## Background

CD73 is a glycosylphosphatidylinositol (GPI) anchored cell surface protein, also known as ecto-5’-nucleotidase (ecto-5’-NT, EC 3.1.3.5). Accumulating data have shown that CD73 play important roles during cancer growth and metastasis [1–3]. CD73 has both enzymatic and non-enzymatic functions [4]. As an enzyme, CD73 catalyzes the AMP breakdown into adenosine. Notably, adenosine is an important purine signaling molecule which has been found to be involved in tumor immunoescape [5]. In addition to its enzymatic function, CD73 is also a regulatory molecule which related to cancer invasive and metastatic properties [3, 6, 7]. Studies have found that CD73 can promote proliferation and migration of several types of cancer cells [6, 8–10]. Cervical cancer, a cancer arising from cervix is due to the abnormal proliferation of cells that have the ability to evade growth suppression. Cervical cancer is high in the rank of cancers affect women, with both the fourth-highest incidence and the fourth-highest fatality rate among women worldwide [11]. Infection with several special types of human papilloma virus (HPV) has been found to be the most high-risk cause of cervical cancer [12]. However, HPV infection was not enough to trigger cervical cancer. Other factors, like molecular alteration have also been found to play important role during cervical cancer development [13, 14]. The role of CD73 in cervical cancer cells has not been well studied. In the present study, we investigated the effect of CD73 overexpression on cervical cancer cells proliferation and migration, and further explored its underlying regulatory mechanisms. Our data demonstrated that CD73 overexpression promoted Hela and SiHa cells proliferation and migration. Moreover, this promotive effect of CD73 should be via an enzymatic activity independent mechanism.

## Methods

### Cell culture

Two human cervical cancer cell lines, Hela and SiHa (American Type Culture Collection, ATCC), were used in this study. The cells were cultured in DMEM medium (Gibco, Carlsbad, NY, USA) supplemented with 10% heat-inactivated Fetal bovine serum (FBS, Sijiqing Biotec, Hangzhou, China) at 37 °C with 5% CO_2_ in a humidified incubator.

### pcDNA-NT5E recombinant expression vector construction and transfection

CD73 coding gene - NT5E was cloned into the pcDNA3.0+ expression vector, then, the pcDNA-NT5E and control plasmids were transfected into Hela and SiHa cells using Lipofecta-mine™ 2000 reagent (Invitrogen, Carlsbad CA, USA) following the protocol offered by the manufacturer. Briefly, parental Hela and SiHa cells (3 × 10^5^/well) were plated in six - well plate and cultured until 70 – 80% confluence. Premixed lipofection and plasmid DNA (10 μl : 4 μg) were added into the wells and incubated for 24 h. And then, the transfected cells were screened by G418.

### CD73 activity assay

The conversion of AMP to adenosine was measured to assess the CD73 activity. Cultured cells were collected and suspended in DHank's buffer at a density of 4 × 10^5^ cells/ml. 500 μl cell suspension was incubated with 1 mM AMP at 37 °C for 1 h, the effect of APCP on CD73 activity was investigated by adding 50 μM APCP into reaction system. Subsequently, cells were centrifuged, and the supernatants (10 μl) were subjected to HPLC analysis (Shimadzu, LC-2030c; column: GL Sciences, C18, 3.5 μm, 2.5 × 250 mm). Adenosine was extracted through a 0-50% methanol/H_2_O phase gradient elution (1 ml/min). The absorbance was measured at 259 nm. The peak volume (PV) for adenosine was calculated using LabSolutions LC Workstation Ver.5.

### MTT cell viability assay

The cells were seeded in 96 - well plate at a density of 4 × 10^3^ cells per well and cultured at 37 °C in DMEM medium plus 10% FBS. At different time point, 10 μl of MTT (5 mg/ml, Amresco, Solon, USA) was added into each well and the cells were incubated for another 4 h at 37 °C. The supernatants were then discarded carefully and 150 μl DMSO was added to each well. The plate was shaken for additional 10 min and the absorbance value was measured at 490 nm by using the microplate reader (Bio-Rad, Hercules, USA). The relative viability of cells was calculated as a percentage using the formula: (mean OD_490_ of treated cells/mean OD_490_ of control cells) × 100%.

### Migration assays

Migration ability of the cells was examined by scratch assay. Transfected and control cells were cultured in six-well plate. The scratch was performed with a pipette tip when cell density reached to 80%. Once scratch was made, cells were gently washed by PBS twice, following cultured with serum-free medium. Images were captured immediately, and 24 h after the scratch was made. The cell migration distance was measured by HMIAS - 2000 software. The migration ability of cells was mirrored by relative migration ratio: Relative migration ratio = (Start distant – End distance)/Start distance.

### Quantitative real-time RT-PCR and western-blot analysis

Quantitative real-time RT-PCR was carried out using the SYBR Green kit (Invitrogen, Carlsbad, USA) according to the manufacturer’s instructions. The relative expression levels of genes were normalized to the endogenous housekeeping gene β-actin. The primer sequence are list in Table 1. For western-blot, the total cell protein was loaded for SDS-PAGE, and then transferred onto polyvinylidene difluoride membranes. Membranes were blocked with 5% non-fat dried milk for 2 h at room temperature, and then incubated with primary antibody (R & D Systems, Inc.) overnight at 4 °C. After washed with TBST for three times, membranes were incubated with secondary antibody for 2 h at room temperature. After washed with TBST, proteins were detected with western blotting luminol reagent (Santa Cruz Biotechnology, Santa Cruz, USA), β-actin were used as the internal standard.

**Table 1:** primer sequence used in the study

Gene name	Forward primer (5’–3’)	Reverse primer (5’–3’)
CD73	GCCTGGGAGCTTACGATTTTG	TAGTGCCCTGGTACTGGTCG
EGFR	CCAAGGCACGAGTAACAA	ACATAACCAGCCACCTCC
VEGF	TTGCCTTGCTGCTCTACCTC	TGCATGGTGATGTTGGACTC
Akt1	ACTGTCATCGAACGCACCTT	TTCTGCAGGACGCGGTTCTC
β-actin	TGACGTGGACATCCGCAAAG	CTGGAAGGTGGACAGCGAGG

### Statistical analysis

All data were confirmed in three biological replicates. The data are expressed as the mean ± standard error. Data comparisons were conducted by using the Student’s *t*-test. *P* < 0.05 was considered to be statistically significant.

## Results

### CD73 overexpression promoted proliferation and migration of cervical cancer cells

**Fig. 1:**
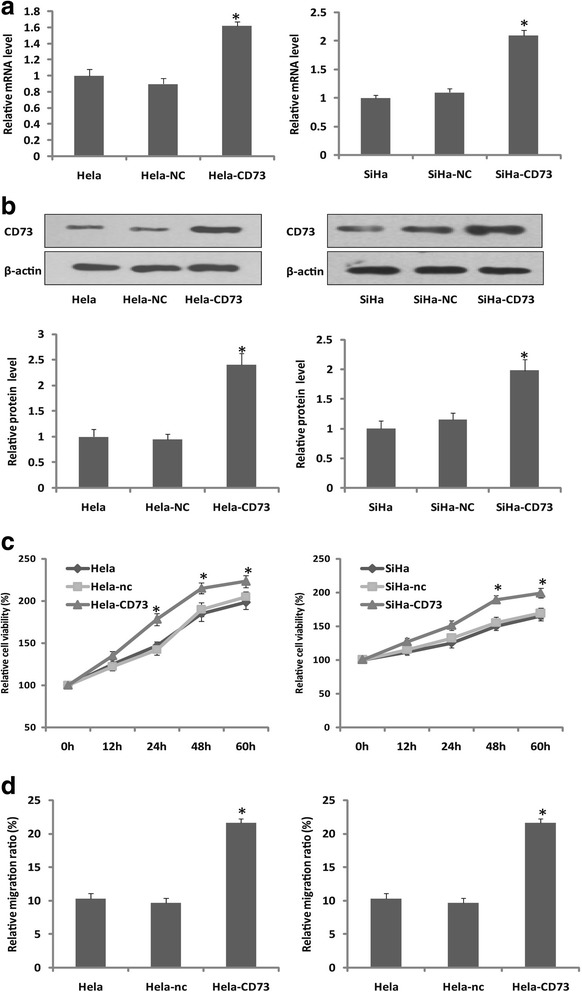
The proliferation and migration of CD73 overexpression cells. **a**, **b** RT-PCR and western-blot showed that CD73 expression in transfected cells (named Hela-CD73 and SiHa-CD73) was significantly high than that in control cells. **c** MTT assay showed that CD73 overexpression promoted Hela and SiHa cells proliferation. **d** Scratch healing assay showed that CD73 overexpression promoted Hela and SiHa cells migration

To determine the effect of CD73 in proliferation and migration of human cervical cancer cells in vitro, the pcDNA-NT5E and control vectors were constructed and transfected into Hela and SiHa cells. The expression level of CD73 in transfected cells was significantly higher than that in mock and parental control cells (Fig. 1a), and the CD73 enzymatic activity was also increased in CD73 overexpressed cancer cells (Additional file 1: Figure S1). Then, the effect of CD73 overexpression on cervical cancer cells proliferation and migration was examined. By using MTT assay, we found that the proliferation rate of CD73 overexpression cells was significantly higher than that of control cells (Fig. 1b). Moreover, in addition to the promotive effect of CD73 on Hela and SiHa cells proliferation, we also found that CD73 overexpression enhanced the Hela and SiHa cells migration ability by using scratch healing assay (Fig. 1c). These results demonstrated that CD73 overexpression promoted cervical cancer cells proliferation and migration in vitro.

### Blocking of CD73 enzyme activity could not revert its promotive effect on cells proliferation and migration

**Fig. 2:**
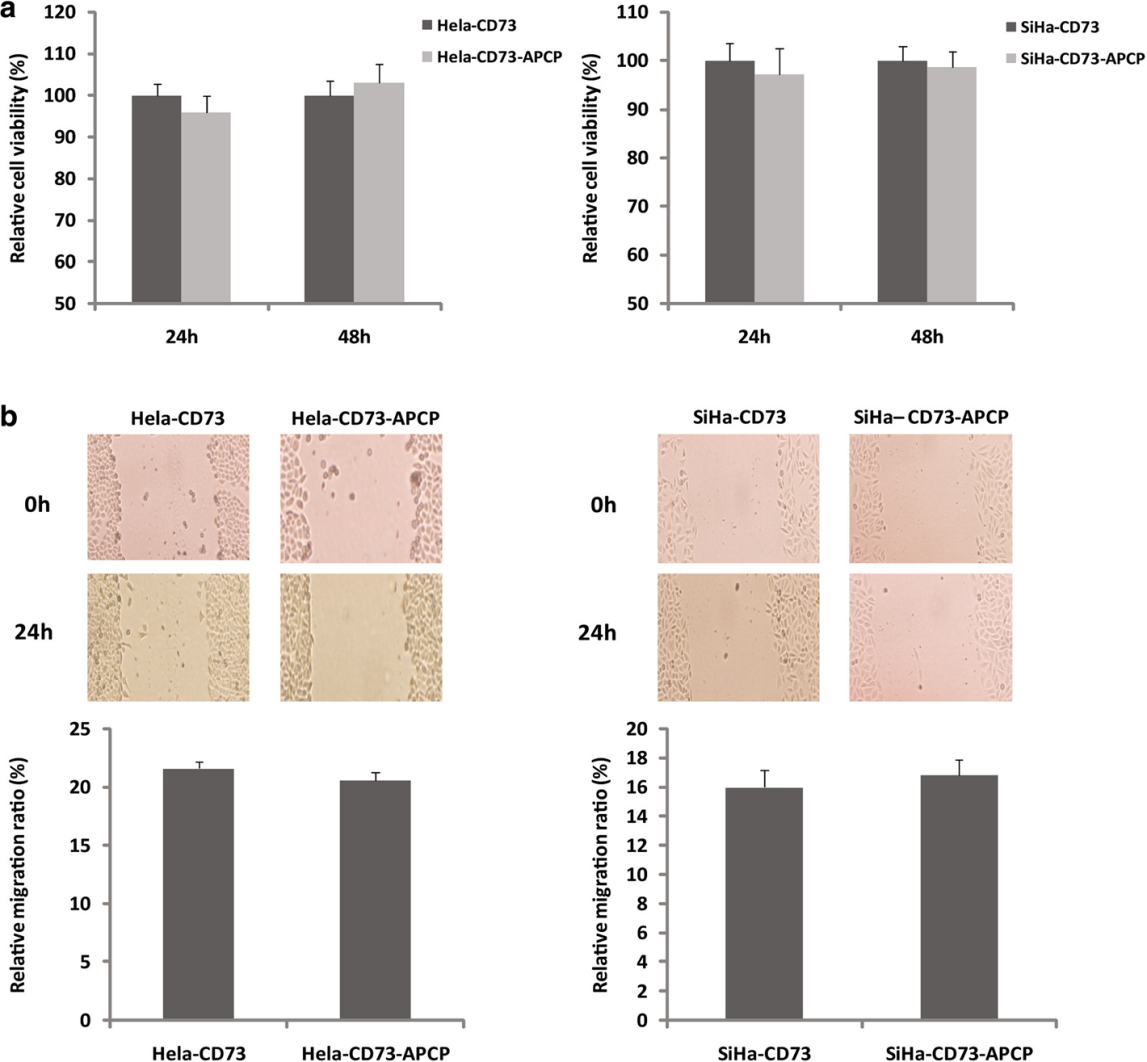
The effect of APCP on CD73 transfected Hela and SiHa cells. **a** MTT assay showed that APCP treatment did not influence the proliferation of CD73 overexpression Hela and SiHa cells. **b** The migration of CD73 transfected cells was not changed in response to APCP treatment. These results suggested that APCP treatment did not revert the promotive effect of CD73 overexpression

CD73 has both enzymatic and non-enzymatic function. To further investigate whether the promotive effect of CD73 on the cells proliferation and migration was dependent on its enzyme activity or was mainly the results of its non-enzymatic function, we used 50 μM APCP (the specific inhibitor of CD73 enzymatic activity) to block the CD73 enzyme activity in the present study. HPLC analysis showed that APCP could inhibit CD73 enzymatic activity significantly. (Additional file 1: Figure S1). Moreover, APCP treatment did not influence the proliferation and migration of CD73 transfected Hela and SiHa cells (Fig. 2), which means that the promotive effect of CD73 is not reverted by blocking of CD73 enzymatic activity. These results indicated that the promotive effect of CD73 overexpression on the proliferation and migration of Hela and SiHa cells was independent of its enzyme activity.

### High concentration of extracellular adenosine decreased cervical cancer cells proliferation and migration

**Fig. 3:**
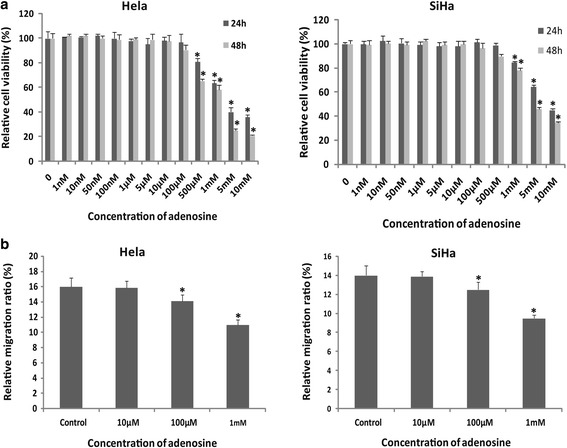
The effect of extracellular adenosine on cells proliferation and migration. **a** Gradient adenosine treated Hela and SiHa cells. The results showed that high concentration of adenosine inhibits cells proliferation. **b** Scratch healing assay showed that high concentration of adenosine diminished the migration of Hela and SiHa cells

As a nucleotidase, CD73 catalyzed AMP breakdown into adenosine. In present study, we examined the effect of adenosine in cervical cancer cells proliferation and migration in vitro. Hela and SiHa cells were treated with different concentrations (1, 10, 50, 100 nM; 1, 5, 10, 100, 500 μM; 1, 5, 10 mM) of adenosine for 24 h and 48 h. After adenosine treatment, MTT assay was used to detect the cancer cells proliferation. As shown in Fig. 3, compared with control cells, the proliferation rate was inhibited by high concentration (>100 μM) of extracellular adenosine in both Hela and SiHa cells, while the cells proliferation was not influenced by low concentration (<100 μM) of adenosine. Moreover, treatment with high concentration of adenosine significantly diminished migration ability of Hela and SiHa cells (Fig. 3). These results suggested that the promotive effect of CD73 overexpression on proliferation and migration in vitro was independent of adenosine (i.e. CD73 enzyme activity).

### CD73 overexpression potentiated EGFR/Akt and VEGF/Akt pathway

**Fig. 4:**
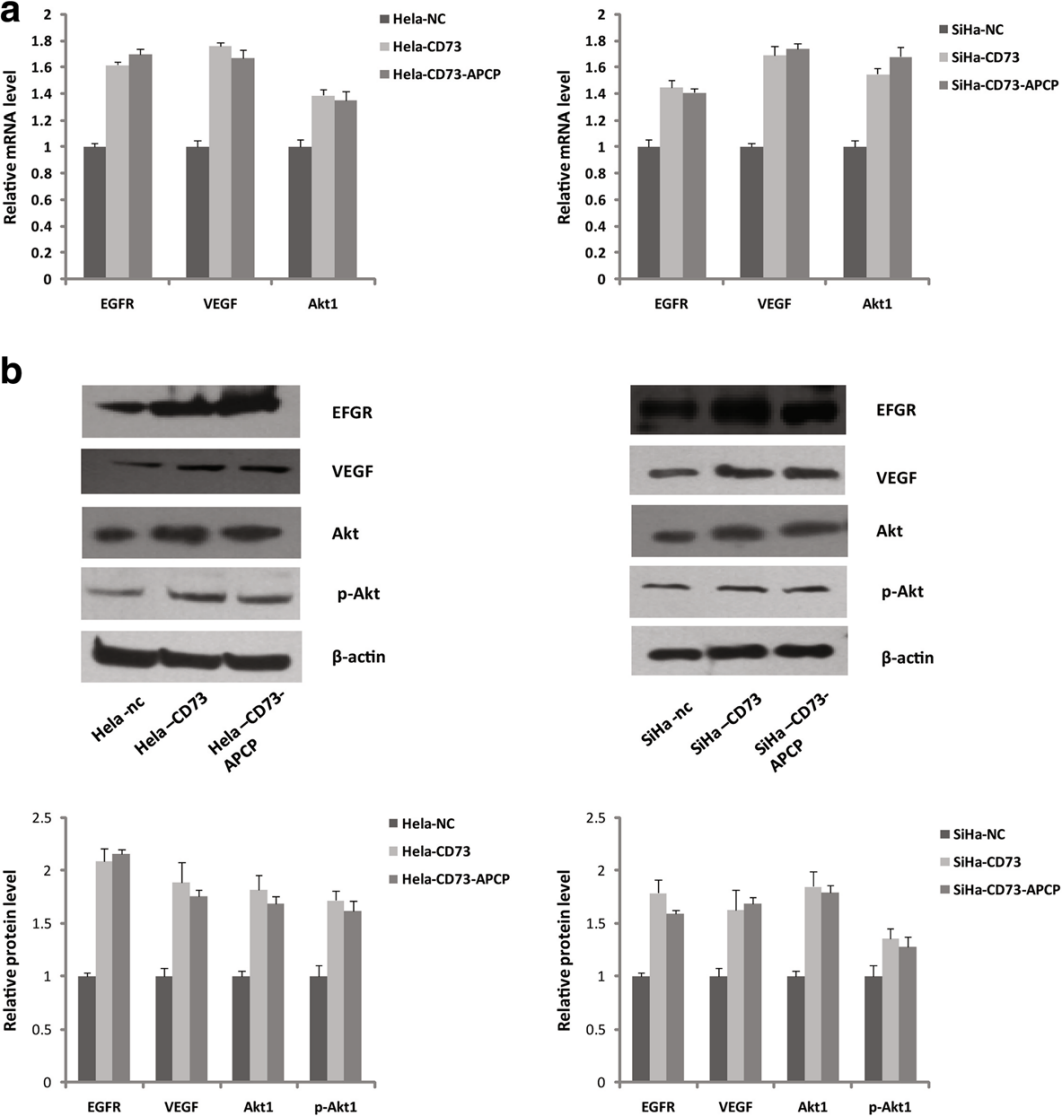
CD73 overexpression potentiated EGFR/Akt and VEGF/Akt pathway. **a** RT-PCR and **b** western-blot showed that the expressions of EGFR, VEGF, Akt, pAkt were significantly higher in CD73 overexpression Hela and SiHa cells in compared with control cells, and moreover, increased effect in CD73 overexpression cells was not reverted by APCP treatment

Since the promotive effect of CD73 on the cells proliferation and migration was independent of its enzyme activity, there should be other mechanism involved in this promotive function. EGFR and VEGF are crucial signaling molecules which have been believed to promote cancer cell growth and metastasis. In this present study, we examined the effect of CD73 overexpression on EGFR and VEGF expression in cervical cancer cells. As shown in Fig. 4, compared with control cells, the EGFR expression was increased by 62% and 45%, while VEGF was increased by 76% and 69% in CD73 transfected Hela and SiHa cells. As Akt is an important downstream signaling molecule of EGFR and VEGF, we also investigated the change of Akt expression in CD73 overexpression cervical cancer cells. The RT-PCR and western-blot results showed that the expression of both Akt and phospho-Akt were significantly increased in CD73 transfected Hela and SiHa cells than that in control cells. These data suggested that CD73 overexpression potentiated EGFR/Akt and VEGF/Akt pathway. Furthermore, to rule out the possibility that these signaling molecules changes were induced by the CD73 enzyme activity, we examined the effect of APCP treatment on EGFR, VEGF and Akt expression. As shown in Fig. 4, the mRNA and protien expressions of these molecules in CD73 overexpression cells were not changed in response to APCP treatment (Fig. 4a, b). And moreover, we also investigated whether adenosine changed these molecules levels by qRT-PCR, the results were negative (Additional file 2: Figure S2). Thus taken together, these data indicated that CD73 ovexpression promoted Hela and SiHa cells proliferation, at least partially, via potentiating the EGFR/Akt and VEGF/Akt signaling pathway, independent of CD73 enzymatic activity.

## Discussion

CD73 has emerged as an important molecule during cancer growth and metastasis. This study was undertaken to evaluate the effect of CD73 on cervical cancer cells proliferation and migration. Our results showed that CD73 overexpression promoted cervical cancer cells proliferation and migration in vitro. CD73 has both enzymatic and non-enzymatic functions in cells. As a nucleotidase, CD73 catalyzes AMP breakdown into adenosine. CD73-generated adenosine has been recognized an important purinergic signaling during cancer progression, which can suppress immune response and assist tumor immune evasion in vivo via multiple pathway [5]. However, besides evading immune destruction, rapid and un-controlled proliferation of tumor cells is another key factor for cancer growth [15]. Regard to this, recent years, researchers investigated the direct effect of extracellular adenosine on cancer cells proliferation in vitro by adding adenosine into culture medium, these data, including ours here, have shown that high concentration of adenosine can inhibit tumor cells proliferation and migration [16–19]. However, this inhibiting effect of high concentration of adenosine is unexpected, since CD73 overexpression has been found to promote tumor cells proliferation and migration in vitro.

At first sight, the promotive effect of CD73 and inhibiting effect of adenosine on cervical cancer cells proliferation and migration seemed to be paradoxical. This apparent discrepancy could be explained on the basis of the following considerations. The concentration of adenosine is determined by a complex ectoenzyme mechanism. The generation and degradation of adenosine is catalyzed by a cascade of enzyme as follows: ATP/ADP breakdown into AMP by CD39, AMP breakdown into adenosine by CD73, and adenosine breakdown into inosine by ADA and its cofactor CD26. Thus, the increased concentration of adenosine by CD73 overexpression may be not enough to inhibit the cancer cells proliferation and migration (the adenosine concentration should be > 100 μM, which can display inhibited effects). As the low concentration of adenosine has no effect on cancer cells proliferation and migration, therefore, the promotive effect of CD73 overexpression on cells proliferation and migration in vitro were independent of its enzyme activity. Indeed, here we proved this hypothesis in Hela and SiHa cells. In present study, we found that the promotive effect of CD73 overexpression on cervical cancer cells proliferation and migration were not reverted by blocking of CD73 enzyme activity. These data suggested that CD73 promoted cervical cancer cells proliferation and migration, which was dependent of its non-enzyme activity. EGFR and VEGF have been believed to promote cancer growth and metastasis [20–23]. Co-expression of CD73 and EGFR or VEGF has been found in other type of cancers [24–26]. Consistently, in this study, we found that EGFR and VEGF expression were markedly increased in CD73 transfected cervical cancer cells. Although Zhi X’s data have suggested that CD73 may promote EGFR expression though regulating some associated transcription factor, such as PPARγ [24]. However, the relevant studies are still few to demonstrate the regulated mechanism of CD73 on expression of EGFR and VEGF. The interaction among them needs further studies. Moreover, the Akt expression, which is an important downstream molecule of EGFR and VEGF, was also significantly higher in CD73 overexpression cells than that in control cells [27]. In the mean while, the EGFR, VEGF and Akt expression in CD73 overexpression cells were not changed in response to APCP treatment. In summary, our data suggest that CD73 overexpression promote cervical cancer cells proliferation and migration, via potentiating EGFR/Akt and VEGF/Akt pathway, independent of its enzymatic activity. These data provide a novel insight into the regulating function of CD73 in cancer and suggest that CD73 may be promising therapeutic target in cervical cancer.

## Conclusions

Our data showed that CD73 promoted cervical cells proliferation and migration, and this promotive effect was independent of CD73 enzyme activity. In addition, we also showed that CD73 overexpression potentiated EGFR/Akt and VEGF/Akt pathway. These data provide a novel insight into the regulating function of CD73 in cancer cells and suggest that CD73 may be promising therapeutic target in cervical cancer.

## Additional files

Additional file 1: Figure S1. CD73 enzymatic activity was assessed by using HPLC analysis. The catalyzed activity was increased in CD73 overexpressed Hela and SiHa cells, while was inhibited by APCP treatment. (JPG 1761 kb)
Additional file 2: Figure S2. 100 μM Adenosine treatment did not change the expression of EGFR, VEGF and Akt. (JPG 335 kb)

## Abbreviations

ATCC: American Type Culture Collection
FBS: Fetal bovine serum
HPLC: High performance liquid chromatography
HPV: Human papilloma virus
PV: Peak volume
qRT-PCR: Quantitative real-time polymerase chain reaction
