# Supplementary figures and images for: CD73 promotes proliferation and migration of human cervical cancer cells independent of its enzyme activity
Source: BMC Cancer. 2017 Feb 15;17:135. doi: 10.1186/s12885-017-3128-5 (PMC5311855; doi:10.1186/s12885-017-3128-5)

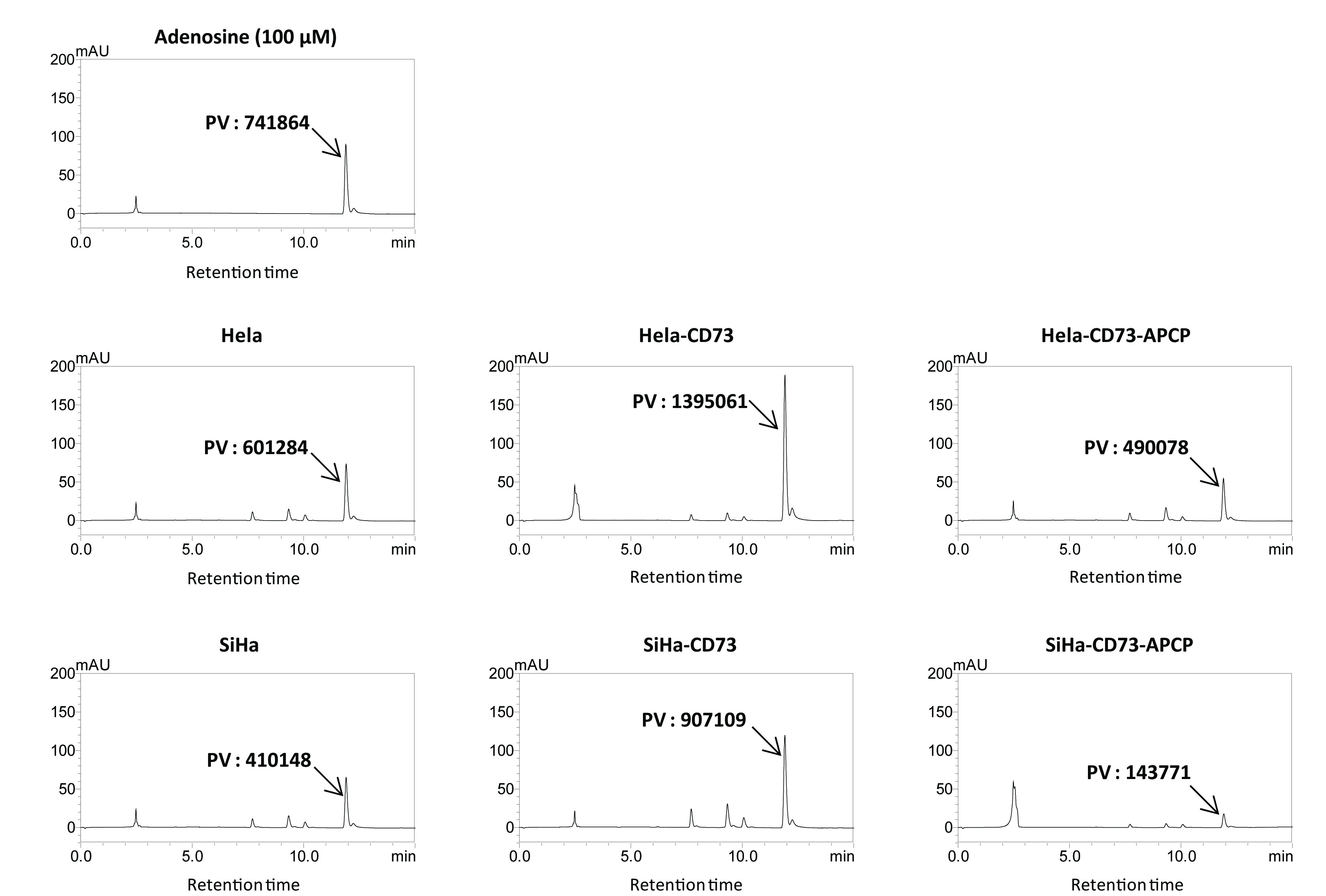

Supplement: Additional file 1: Figure S1. — CD73 enzymatic activity was assessed by using HPLC analysis. The catalyzed activity was increased in CD73 overexpressed Hela and SiHa cells, while was inhibited by APCP treatment. (JPG 1761 kb) [file 12885_2017_3128_MOESM1_ESM.jpg]

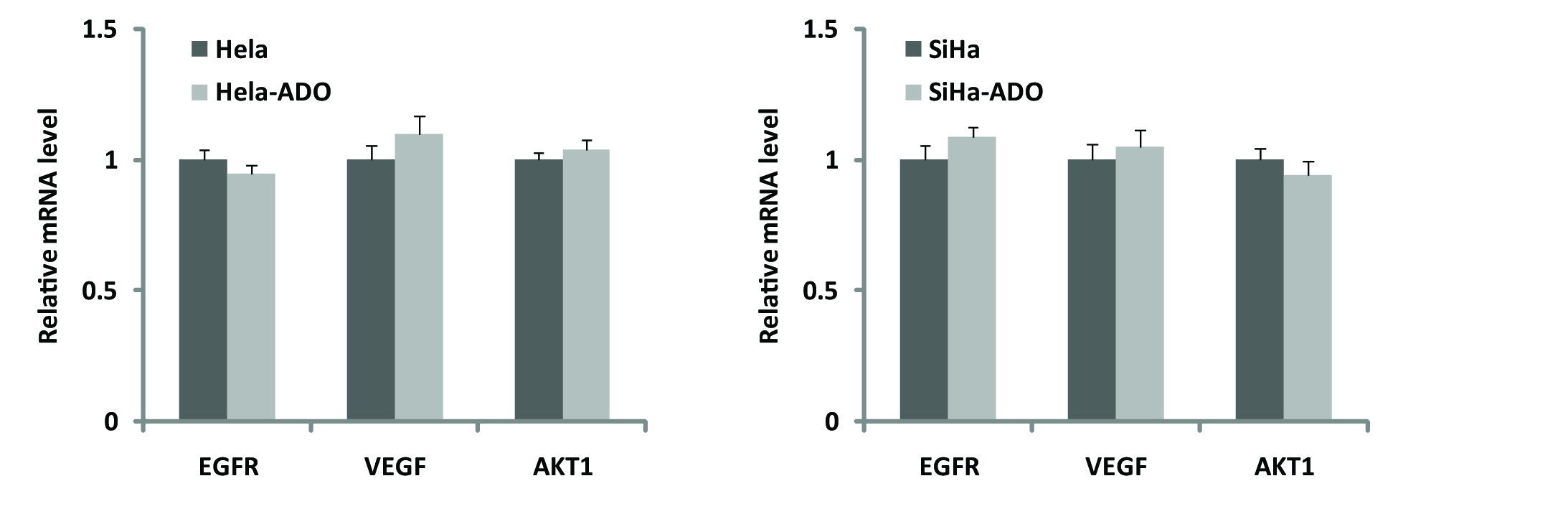

Supplement: Additional file 2: Figure S2. — 100 μM Adenosine treatment did not change the expression of EGFR, VEGF and Akt. (JPG 335 kb) [file 12885_2017_3128_MOESM2_ESM.jpg]
